# Reversible splenial lesion syndrome (RESLES) due to acute intermittent porphyria with a novel mutation in the hydroxymethylbilane synthase gene

**DOI:** 10.1186/s13023-020-01375-y

**Published:** 2020-04-19

**Authors:** Jing Yang, Fei Han, Qianlong Chen, Tienan Zhu, Yongqiang Zhao, Xuezhong Yu, Huadong Zhu, Jian Cao, Xiaoqing Li

**Affiliations:** 1 Emergency Department, Peking Union Medical College Hospital, Chinese Academy of Medical Sciences and Peking Union Medical College, Beijing, China; 2 Department of Neurology, Peking Union Medical College Hospital, Chinese Academy of Medical Sciences and Peking Union Medical College, Beijing, China; 3 State Key Laboratory of Cardiovascular Disease, Beijing Key Laboratory for Molecular Diagnostics of Cardiovascular Diseases, Diagnostic Laboratory Service, Fuwai Hospital, National Center for Cardiovascular Diseases, Chinese Academy of Medical Sciences and Peking Union Medical College, Beijing, China; 4 Department of Hematology, Peking Union Medical College Hospital, Chinese Academy of Medical Sciences and Peking Union Medical College, Beijing, China; 5 Department of Radiology, Peking Union Medical College Hospital, Chinese Academy of Medical Sciences and Peking Union Medical College, Beijing, China; 6 Department of Gastroenterology, Peking Union Medical College Hospital, Chinese Academy of Medical Sciences and Peking Union Medical College, Beijing, China

**Keywords:** Acute porphyria, Reversible splenial lesion syndrome, Gene mutation, Hyponatremia

## Abstract

**Background:**

Reversible splenial lesion syndrome (RESLES) is a clinico-radiological syndrome characterized by the presence of reversible lesions specifically involving the splenium of the corpus callosum (SCC). The cause of RESLES is unknown. However, infectious-related mild encephalitis/encephalopathy (MERS) with a reversible splenial lesion remains the most common cause of reversible splenial lesions. Acute intermittent porphyria (AIP) is an autosomal dominant disorder caused by a partial deficiency of porphobilinogen deaminase (PBGD), the third enzyme in the heme biosynthetic pathway. It can affect the autonomic, peripheral, and central nervous system.

**Result:**

In this study, we report a 20-year-old woman with AIP who presented with MRI manifestations suggestive of RESLES, she had a novel HMBS nonsense mutation, a G to A mutation in base 594, which changed tryptophan to a stop codon (W198*). Conclusion: To the best of our knowledge, this is only one published case of RELES associated with AIP.

## Introduction

Acute intermittent porphyria (AIP) is a rare autosomal dominant disorder affecting heme biosynthesis. AIP is caused by a partial deficiency of porphobilinogen deaminase (PBGD) (alternative name hydroxymethylbilane synthase (HMBS)), which is the third enzyme in the heme biosynthetic pathway. The presentation of AIP is highly variable and nonspecific and can involve the autonomic, peripheral and central nervous systems [1, 2]. Reversible splenial lesion syndrome (RESLES, sometimes also named MERS), first identified by Tada et al. [3], is a clinico-radiological syndrome characterized by transient splenial lesions with high signal intensity on T2-weighted images (T2WI), fluid-attenuated inversion recovery images (FLAIR), and diffusion-weighted images (DWI) and hyperisointense signals on T1-weighted imaging (T1WI) sequences without contrast enhancement [4]. The exact pathophysiology of RESLES is unknown, and it has been associated with several disorders of varied origin, including infection, high-altitude cerebral edema, seizures and antiepileptic drug (AED) withdrawal, and metabolic disturbances [4–6]. To the best of our knowledge, there are no reports to date of reversible splenial lesions associated with AIP. Here, we described an AIP case representing RESLES, which was confirmed by genetic testing of HMBS.

## Materials and methods

### Case report

**Fig. 1:**
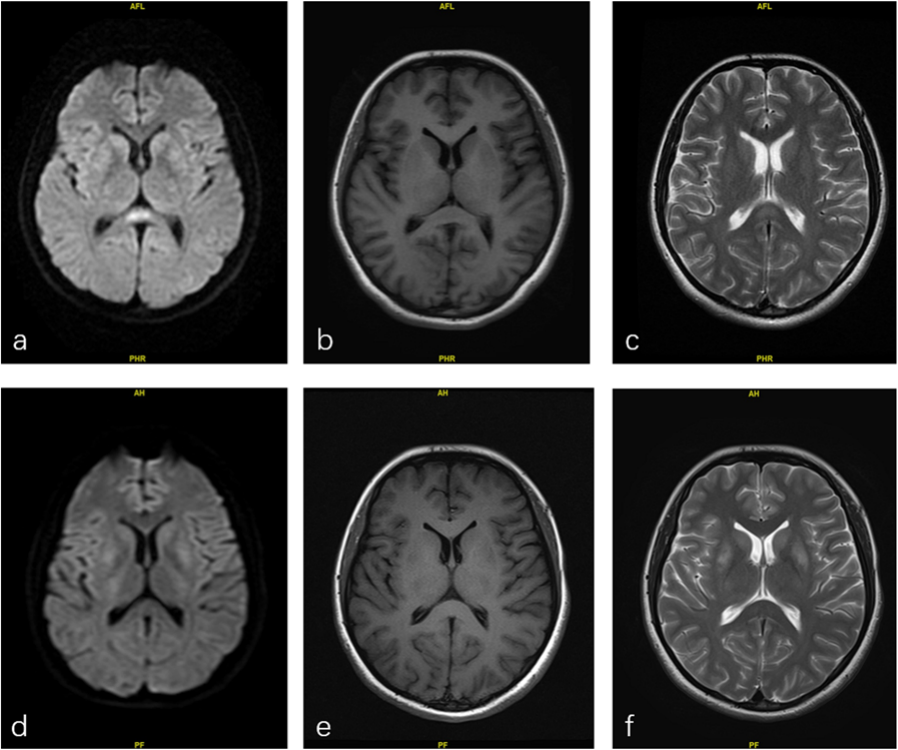
The lesion in the midline of SCC was hyperintensity on DWI **a** and T2WI **c**, isointense signals on T1WI **b** (2019-7-17). Follow-up (2019-8-9) images show complete resolution **d**, **e**,**f**)

A 20-year-old Chinese Han woman, previously well, presented to the hospital on 9 July 2019 with severe continuous abdominal pain, nausea, vomiting and dark tea-coloured urine. She was diagnosed with an “intestinal obstruction” and treated in a local hospital for a few days. Her serum sodium concentration was lower than normal (121.9 mmol/l↓). She became sleepy, confused and convulsive on 12 July. On 13 July 2019, the patient was transferred to the emergency department of our hospital, and her serum sodium concentration was decreased to 108 mmol/L. Biochemical tests were positive for urine porphobilinogen (PBG) and negative for free erythrocyte protoporphyrin and urine uroporphyrin, establishing the diagnosis of AIP. Her urine osmolality was normal (119 mOsm/kgH2O) when plasma osmolality was lower than normal (249 mOsm/kgH2O). However, due to the lack of hemin in China, only supportive treatments could be administered. After 4 days of treatment with 250 g of intravenous glucose per day and fluid restriction (<2000 ml per day), with Tolvaptan 3.5 mg once a day for 3 days, she recovered consciousness, and her serum sodium concentration was gradually increased to 135 mmol/L. Her initial brain magnetic resonance imaging results on July 17 revealed an isolated lesion of the SCC, with T2 and FLAIR hyperintensity, T1 hypointensity, and corresponding reduced values on apparent diffusion coefficient (ADC) maps. (a, b and c of Fig. 1), whereas her cerebral spinal fluid (CSF) testing results were almost normal. The cranial MRI performed two weeks later revealed that the lesions determined on the first MRI were significantly regressed (d, e and f of Fig. 1, respectively).

On the basis of these findings, we decided to examine the genetic causes of the disease in her family.

### Genetic testing of the HMBS gene

The molecular genetic test was performed by direct sequencing of the HMBS gene to confirm acute intermittent porphyria. All 14 exons of the HMBS gene and a minimum of 20 base pairs of intronic DNA flanking of each exon were amplified by polymerase chain reaction (PCR) (Tiangen Biotech, Beijing, China) and subsequently sequenced using the BigDye Terminator Cycle Sequencing Kit V 3.1 (ABI Biosystems) on an ABI PRISM 3730 Genetic Analyzer, according to the manufacturer’s directions.

## Results

**Fig. 2:**
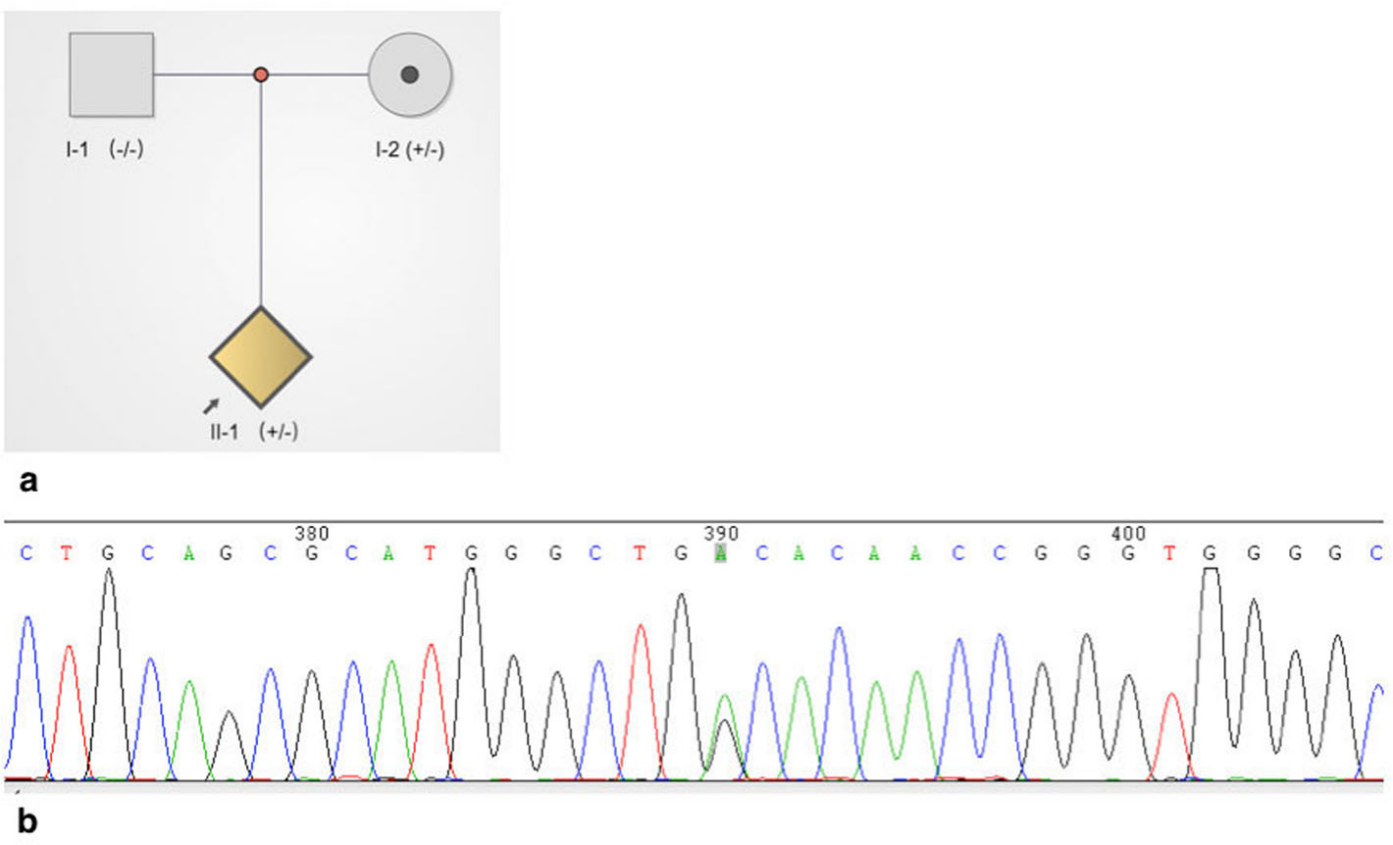
A novel HMBS gene nonsense mutation was identified. **a**. pedigree with HMBS gene mutation (The arrow indicated the proband); **b**. A novel HMBS gene mutation c.594G > A (W198*) was identified in the proband and her mother

A novel HMBS gene (NM_000190) nonsense mutation, c.594G > A (W198*), was detected by Sanger sequencing in the proband, which led to a premature termination codon (Fig. 2). After screening her family members, we found that her mother also carried the same mutation, while her father did not (Fig. 2).

## Discussion

In 2004, Tada et al. [3] reported a series of 15 patients with mild encephalitis/encephalopathy with a reversible splenial lesion (MERS). In 2011, Garcia-Monco et al. [5] reviewed the MEDLINE database from 1966 to 2007 and termed the presence of transient lesions involving SCC reversible splenial lesion syndrome (RESLES). Therefore, at times, RESLES associated with encephalitis/encephalopathy was interchangeably termed MERS [5, 7]. RESLES is characterized by reversible lesions in the central portion of the splenium of the corpus callosum (SCC) [3, 8]. RESLES is most often identified in patients with seizures and/or antiepileptic drug withdrawal [9, 10], and it is also associated with infections of various pathogens, such as influenza virus, rotavirus, measles, herpesvirus 6, adenovirus, mumps, Epstein-Barr virus, Escherischia coli, and others [11–13]. Typically, the clinical neurological symptoms of RESLES include mildly altered states of consciousness, delirium, and seizures after a range of previous viral infections but usually have complete recovery without neurological sequelae after a short disease course [7, 14].

There are few reports in the literature on MRI findings of porphyria cases with central nervous system (CNS) involvement [15, 16]. Many of studies suggested that MRI changes are related to posterior reversible encephalopathy syndrome (PRES) [17–19]. In this report, we have presented a new MRI finding of AIP that has not been previously reported.

According to Hoshino et al. [20], the diagnostic criteria of RESLES (MERS) are as follows: (1) clinical onset associated with neuropsychiatric symptoms, such as impaired consciousness within 1 week after fever onset; (2) complete recovery without sequelae, mostly within 10 days after the onset of neuropsychiatric symptoms; (3) high-signal-intensity lesion in the SCC; (4) involvement of the entire corpus callosum and bilateral cerebral white matter with symmetrical pattern; (5) lesion disappearance within 1 week, with no residual signal changes or atrophy. Despite our proband undergoing the second MRI 15 days after the first one and without fever, she fulfils this diagnostic criterion. RESLES is classified as RESLES type I or RESLES type II, depending on the involvement of SCC alone or other white matter areas. Our patient was a RESLES type I case.

Acute intermittent porphyria (AIP) is one of four forms of acute porphyria that is caused by an inherited deficiency of PBGD, which catalyses the third enzymatic step in the biosynthesis of heme. Symptoms in AIP, which occur as intermittent attacks and may be life-threatening, are caused by excess production of porphyrin precursors on the visceral, peripheral, autonomic, and central nervous systems [1]. Clinical manifestations of CNS involvement include epileptic seizures, impaired consciousness, behavior changes and hyponatremia caused by inappropriate antidiuretic hormone syndrome [21, 22]. The symptoms of our proband included abdominal pain, nausea, vomiting, confusion, delirium, seizures and hyponatremia.

The reason for the transiently reduced diffusion within the lesions on MRI is still unknown, which has been suggested to be due to hypotonic hyponatremia or a myelin-specific neurotoxin released by a pathogen [6, 23]. The possible cause of hyponatremia of RESLES is SIADH, which is also considered a cause of hyponatremia in patients with AIP [22]. Since she had no evidence of infection, hyponatremia may be a contributing factor of RESLES in our proband. However, urine and blood osmolality of our proband were both lower than normal, perhaps because she had taken 0.25 mg tolvaptan 12 h before. Tolvaptan could significantly decrease the urine osmolality [24, 25]. Tolvaptan did not exacerbate symptoms in the acute phase of AIP in our proband, and perhaps it is safe to use of tolvaptan in AIP with hyponatremia.

In mainland of China, laboratory investigations (erythrocytic PBG deaminase levels, urinary, fecal, and plasma porphyrin levels) are unavailable. Molecular genetic testing provides a precise diagnosis to differentiate AIP from other acute porphyrias and can then be used to identify AIP in relatives of the proband. In this study, we identified a novel HMBS gene mutation (W198*) in a Chinese family. Most cases of RESLES have occurred in children in East Asian populations, mostly Japan, and there has also been one case report of sisters with RESLES, which would support the genetic vulnerability hypothesis [26]. AIP is a hereditary disease, suggesting that a genetic factor might be involved in some RESLES patients. This is the first reported case of RESLES following AIP, and she had a novel HMBS nonsense mutation. Our case report widens the phenotype of the neurological manifestations associated with AIP. Since porphyria is a rare disease, the major limitation of our work was the small sample size. Further clinical, radiological and genetic studies of RESLES are necessary for a definite conclusion.

## Conclusion

In conclusion, we report the first reported case of RESLES following AIP with a novel HMBS nonsense mutation. Hyponatraemia may be a contributing factor of RESLES.

## Data Availability

The data used and/or analysed to support the results of the current study are available from the corresponding author on reasonable request.

## Abbreviations

RESLES: Reversible Splenial Lesion Syndrome
MERS: Mild Encephalitis/Encephalopathy with Reversible Splenial Lesion
AIP: Acute Intermittent Porphyria
PBGD: Porphobilinogen deaminase gene
SCC: Splenium of the corpus callosum
HMBS: Hydroxymethylbilane synthase
FLAIR: Fluid-attenuated inversion recovery images
T2WI: T2-weighted images
T1WI: T1-weighted images
ADC: Apparent diffusion coefficient
CSF: Cerebral spinal fluid
PCR: Polymerase chain reaction
